# Applications of mobile learning on college students’ water leisure sports in the post-pandemic era

**DOI:** 10.3389/fpsyg.2022.1011551

**Published:** 2022-10-11

**Authors:** Ko-Chia Chen, Shi-Jer Lou, Chih-Cheng Tsai

**Affiliations:** ^1^Department of Tropical Agriculture and International Cooperation, National Pingtung University of Science and Technology, Pingtung, Taiwan; ^2^Graduate Institute of Technological and Vocational Education, National Pingtung University of Science and Technology, Pingtung, Taiwan; ^3^Department of Industrial Technology Education, National Kaohsiung Normal University, Kaohsiung, Taiwan

**Keywords:** mobile learning, water leisure sports, post-pandemic, college students, TAM, PLS-SEM

## Abstract

This study explored the relationship and influence of college students’ participation in water leisure sports, as well as the technology acceptance model (TAM). With the rapid development of the economy, the government is promoting various water leisure sports centered on the concept and policy of a maritime- and ocean-based nation. Based on the TAM, this study investigated the relationships among its ease of use, usefulness, water leisure involvement, benefits, barriers, and intentions to participate in water activities in connection with college students participating in water leisure sports. A total of 420 college students who participated in water leisure activities were sampled. There were 370 valid questionnaires, and the recovery rate of valid questionnaires was 82.2%. The data were analyzed by the structural equation modeling of the partial least squares method (PLS-SEM). The results show that the ease of use of water facilities had a positive effect on the usefulness, involvement, and participation in water activities; the usefulness of water facilities had a positive and significant impact on the intention to participate in water activities; water leisure involvement had a positive and significant impact on the benefits and the intention to participate in water activities; the intention to participate in water activities had a positive and significant impact on the benefits of water leisure activities. Furthermore, the study found that the intention to participate in water activities had a mediating effect between water leisure involvement and water leisure benefits; water leisure involvement had a mediating effect between the ease of use of water facilities and the intention to participate in water activities; the usefulness of water facilities had a mediating effect between the ease of use of water facilities and the intention to participate in water activities. In addition, the interaction between water leisure involvement and water leisure constraints had an interfering effect on water leisure benefits. Accordingly, recommendations for promotion and implementation are provided. Based on the TAM, the study provided suggestions for implementing water leisure sports to promote college students’ participation behavior in water leisure sports.

## Introduction

### Research background and motives

With the economy’s progress, work has become more stressful, impelling people to pay more attention to leisure activities to relieve pressure after work. In addition to relaxing and relieving stress, leisure activities have become a basic need of modern life (Huang, 2000), which implies time, psychology, spirit, experience, activity, and freedom. Roberts (2006) leisure activities can enhance the sense of purpose and pursuit of aspiration, thus describing personal feelings, wild imagination, and the commitment to learning new skills and creating pleasant pastime and practice in daily life.

Amidst the advancement of technology and the improvement of living standards, leisure has become the most important and frequent activity among Chinese individuals. Such an increase in leisure awareness has improved the proportion and time spent on leisure activities.

According to Katz (1996) most sports and leisure activities in which individuals are actively involved are water-related. In addition, surrounded by seas, Taiwan enjoys unique and abundant water resources, including fast-flowing streams, charming lakes, vibrant wetlands and marshes, and diverse marine ecology compared to other countries. Choi and Park (2021) argued that water leisure activities can fulfill the harmony between humans and nature, which presents human’s stamina to overcome nature; furthermore, modern people who prefer natural activities can understand that humans are a part of nature better through harmony. The term “water” is a broad term that covers all areas where there is water, and the definition of water activities refers to water-related activities that are performed through human body movements or special equipment in specific areas of water, air, and land adjacent to water (Sung et al., 2010).

Since the outbreak of COVID-19, outdoor activities and physical courses have been suspended or conducted online. Hence, continuing to promote water leisure sports in the post-pandemic era is a test for individuals who are into water activities. The spread of information technology and mobile devices, such as smartphones, tablets, laptops, and wearable devices, has led to the transformation in learning and the accelerated entry into a new era of mobile learning. The rampage of COVID-19 has changed human lifestyle and affected more than 91% of learners worldwide. Nevertheless, the cancelation is replaced by the argument that “Leisure involvement refers to the understanding of the meaning of leisure to life and relevant leisure behaviors” (Pan et al., 2018). The application of mobile learning brings new opportunities to change the learning process and provides convenient and flexible learning channels for students (Abbasi et al., 2020). Mobile learning is an extension of digital learning in which learners can access information and learn without the constraints of time and space with the aid of miniaturized mobile devices. It implies learning through electronic devices using social and content interaction in multiple contexts, allowing learners to interact with their peers, educators, experts, and the world beyond the constraints of time and space (Bernacki et al., 2020). Hence, mobile learning transformed the traditional teaching curriculum and increased the freedom and convenience of learning. In this study, online videos of water-related activities were used as a pedagogical aid during the pandemic to explore the learning benefits of mobile learning applied to water leisure sports.

Havitz and Dimanche (1990) suggested that leisure involvement affects visitors’ experience of the destination and the perceived risk during recreation. The level of leisure involvement is a common and important issue in studies examining the influence of subjective factors on leisure effectiveness. Solomon (2011) defined involvement as “the degree of perceived personal importance, and the interest aroused by a stimulus in a given context.” In addition, scholars have pointed out that individuals’ preferences and participation in leisure activities may be influenced by a number of factors, such as leisure constraints. Leisure constraints influence individuals’ preferences and participation in activities in various ways (Crawford and Godbey, 1987). Henderson et al. (1988) who defined leisure as “all factors interfering with satisfying leisure activities and leading to a pause in these activities, or complex factors affecting leisure activities and causing different choices” and “Kim et al. (2022) who argued that some leisure barriers come suddenly, other than existing interference factors”. For example, COVID-19 has limited the social structure and temporarily torn people away from leisure activities. Thus, a “leisure constraint” refers to any factor that interferes with or interrupts an individual’s leisure preference and influences an individual’s subjective perception of dislike or inability to engage in leisure activities successfully. However, a barrier does not absolutely negatively affect an individual’s participation in leisure activities.

DeNora (2000) referred to the physical and mental health and enrichment experiences gained from leisure activities as leisure benefits. Bammel and Burrus-Bammel (1982) considered leisure benefits as the benefits individuals obtain from participating in leisure activities. Benefits are the beneficial effects of various aspects of an individual’s participation in an event, which satisfy internal and personal needs and interaction with other participants. In terms of leisure benefits, the benefits gained from participating in any leisure sport are not only the achievement of a goal but also the benefit received for the goal (Hsu and Chen, 2020).

What is the relationship between leisure involvement and the participants’ level of intention in water activities? Is the degree of leisure benefits evident? Does the higher level of obstruction to participants’ water leisure activities lead to a higher level of obstruction to the benefits of participation? Do leisure involvement, leisure benefits, and leisure constraints affect participants’ participatory behaviors in post-pandemic leisure activities conducted through mobile learning? These are the questions that were investigated in this study.

### Research purpose

Lin et al. (2020) who found that the difficulties students have in adopting mobile learning relate to the perceived ease of use and perceived usefulness proposed by the Technology Acceptance Model and Hu et al. (2022) who noted that the effectiveness of online learning can be evaluated according to the extent that students perceive the usefulness and ease of use of online learning, while TAM assumes that the extent of students accepting online learning is determined by their behavior intention that can be directly explained by perceived usefulness and perceived ease of use, therefore in this study, the technology acceptance model (TAM) was used to explore the participation behavior of college students in water leisure sports to understand the relationships among ease of use, usefulness, intentions, benefits, participation in water leisure activities, and barriers. The research purposes are as follows:

1. To explore college students’ perceived usefulness, ease of use, intention, involvement, benefits, and barriers to participation in water leisure activities;
2. To investigate the correlation between the ease of use and usefulness of water facilities, intention, benefits, involvement, and barriers;
3. To explore the mediating effects of the usefulness of water facilities, involvement, and intention;
4. To explore the interference effect of water leisure involvement on water leisure constraints, intentions, and benefits.

## Research method

### Research subjects and area

This study examined the relationship between college students’ participation behavior and the technology acceptance model (TAM) in water leisure sports. The research subjects were college students who had participated in water leisure sports. The limitations of this study are as follows. Only students who participated in water leisure sports were surveyed, and complete information about the participants’ backgrounds could not be provided. The quantitative survey was limited by the perception of the participants’ answers, and it was assumed that they filled in detailed answers. Only the relationships among the ease of use, usefulness, intention to participate, involvement, benefits, and barriers to participation were explored to understand the participation behavior of college students engaging in water leisure activities. With a cross-sectional research design, it was difficult to infer causal relationships among the studied variables.

**FIGURE 1:**
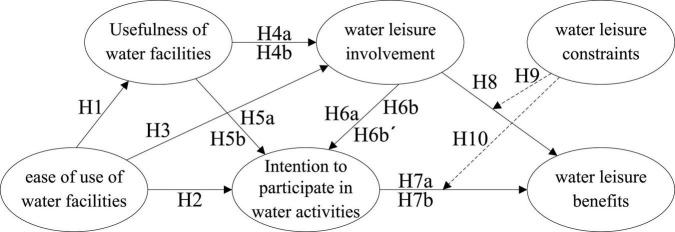
Conceptual model structure.

Furthermore, this study used the TAM as a prototype. Based on the research purposes, the model was revised to align with the relationship between college students’ participation behavior when engaging in water leisure activities and the TAM. The structure is shown in Figure 1. The research hypotheses to be verified are as follows:

H1: The ease of use of water facilities has a positive effect on the usefulness of water facilities.

H2: The ease of use of water facilities has a positive effect on the intention to participate in water activities.

H3: The ease of use of water facilities has a positive effect on water leisure involvement.

H4a: The usefulness of water facilities has a positive effect on water leisure involvement.

H4b: Water leisure involvement has a mediating effect on the usefulness of water facilities and the intention to participate in water activities.

H5a: The usefulness of water facilities has a positive effect on the intention to participate in water activities.

H5b: The usefulness of water facilities has a positive effect on the ease of use of water facilities and the intention to participate in water activities.

H6a: Water leisure involvement has a positive effect on the intention to participate in water activities.

H6b: Water leisure involvement has a mediating effect on the ease of use of water facilities and the intention to participate in water activities.

H6b′: Water leisure involvement has a mediating effect on the usefulness of water facilities and the intention to participate in water activities.

H7a: The intention to participate in water activities has a positive effect on water leisure benefits.

H7b: The intention to participate in water activities has a mediating effect on water leisure involvement and water leisure benefits.

H8: Water leisure involvement has a positive effect on water leisure benefits.

H9: Water leisure constraints have an interfering effect on water leisure involvement and water leisure benefits.

H10: Water leisure constraints have an interfering effect on the intention to participate in water activities and water leisure benefits.

### Research tools

The structural equation modeling of the partial least square method (PLS-SEM) was used in this study to quantify and analyze the sample of college students in Taiwan. The questionnaire of Zhuang et al. (2021), comprising six questions, was used as a reference for the analysis of the ease of use and usefulness of water facilities; the questionnaire of Fichten et al. (2016), comprising six questions, was used as a reference for the analysis of the intention to participate in water activities; the questionnaires of Mclntyre and Pigram (1992), Kim et al. (1997), and Kyle and Mowen (2005), comprising 18 questions, were used as a reference for the analysis of water leisure involvement; the questionnaire of Chiang (2011), comprising 23 questions, was used as a reference for the analysis of leisure benefits; the questionnaire of Raymore et al. (1993), comprising 18 questions, was used as a reference for the analysis of water leisure constraints. The questions were compiled to form the Technology Acceptance Model of the Water Leisure Sports Participation Scale. The 5-point Likert scale was used to measure levels of agreement, namely, “strongly agree,” “agree,” “fair,” “disagree,” and “strongly disagree.”

### Data processing

The partial least squares (PLS) method was used in this study for the conceptual model test. PLS is a structural equation modeling (SEM) analysis technique in performing principal component analysis of structural equation models based on the assumptions of least square methods. Since PLS does not require the data to be under the assumption of multivariate normal distribution, there is no need to test the data for the hypothesis of normal distribution. It can also handle both formative and reactive dimensions, giving it the advantage of analyzing complex predictive models (Chin and Newsted, 1999). PLS can test both measurement and structural models and overcome the problem of collinearity derived from multiple variables. In particular, SmartPLS, developed by Ringle et al. (2005), was used as an analytical tool in this study. The analysis and estimation steps of PLS were divided into two stages: the first stage involved analyzing the reliability and validity of the measurement model, and the second stage involved estimating and validating the path coefficients and explanatory power of the structural model by the repeated indicator method for the second-order conformal research model.

#### Measurement model analysis

The measurement model sets the relationship between the potential and the observed variables to establish the relationship between the measurement indicators and the potential variables and examine the reliability and validity of the measurement indicators (i.e., questionnaires and scales) through the confirmatory factor analysis. The reliability analysis tools include the loading of items, Cronbach’s α value, composite reliability (CR), and average variance extracted (AVE) of the potential variables. Cronbach’s α value and CR for each dimension must reach 0.7 (Bagozzi and Yi, 1988; Nunnally and Bernstein, 1994; Hair et al., 2016) and AVE 0.5 (Bagozzi and Yi, 1988) to have high reliability and validity. Validity includes convergent validity and discriminant validity. The criteria for convergent validity require each item to have a higher loading in its corresponding dimension than the other dimensions, and it should be greater than 0.7 (Bagozzi and Yi, 1988; Hair et al., 2006). On the other hand, the criteria for discriminant validity require the square root of AVE of each dimension to be greater than the correlation coefficient (F) of the corresponding dimension (Fornell and Larcker, 1981), and the correlation coefficient should be less than 0.71 (MacKenzie et al., 2011).

#### Structural equation modeling

A structural model describes the causal relationship between many variables. The structural equation modeling (SEM) consists of a measurement model and a structural model. The measurement model considers the appropriateness of each measure indicator for interpreting dimensions. Given that the structural model can specify the relationships among dimensions, it was used to examine the assumptions of the structure of this study. A systematic approach was proposed by Hair et al. (2016) to evaluate the structural model, which is divided into stages, namely, collinearity diagnosis of the structural model, significance check of the path coefficients, evaluation of R2 size, and assessment of the explanatory effect value f2. In general, the explanatory power of a model is considered weak when the R2 value is close to 0.25, moderate when the R2 value is close to 0.50, and significant when the R2 value is close to 0.75 (Hair et al., 2016). The f2 value proposed by Cohen (1988) is used to assess whether the external derivative variable has significant explanatory power for the internal variables. Its principles are as follows: it is considered a small effect when 0.02 < f2≦0.15, a medium effect when 0.15 < f2≦0.35, and a large effect when f2 > 0.35.

## Results analysis

### Basic data analysis of samples

The study was conducted from 1 February 2022 to 31 May 2022. A total of 420 questionnaires were collected, and there were valid 370 questionnaires after deducting invalid questionnaires, with a valid return rate of 88%. The sample analysis data are shown in Table 1.

**TABLE 1:** Basic information background analysis.

Variable	Category	Percentage	Variable	Category	Percentage
Gender	Male	52%	Age	18 years old	0%
	Female	48%		19 years old	17%
Amount spent on leisure activities per month	Below NTD 1,000	36%		20 years old	25%
	NTD 1,001∼3,000	12%		21 years old	38%
	NTD 3,001∼5,000	27%		22 years old	14%
	NTD 5,001∼7,000	8%		23 years old or older	6%
	NTD 7,001 and above	17%	Year in College	Freshman	0%
Annual household income	Below NTD 500,000	33%		Sophomore	26%
	NTD 510,000∼700,000	21%		Junior	31%
	NTD 710,000∼900,000	18%		Senior	39%
	NTD 910,000∼1,100,000	13%		Student of extended study	2%
	NTD 1,110,000 and above	15%		Master/doctoral program	2%
Department and school	Department of Agriculture	0%	Accommodation	Living with a family member	40%
	Department of Science and Technology	4%		Off-campus apartment	32%
	Department of Management	14%		School dormitory	28%
	Department of Humanities and Social Sciences	25%	Domicile	Northern Taiwan (Taipei, New Taipei, Keelung, Taoyuan, and Hsinchu)	25%
	International College	1%		Central Taiwan (Miaoli, Taichung, Changhua, Nantou, and Yunlin)	8%
	Department of Veterinary	0%		Southern Taiwan (Chiayi, Tainan, Kaohsiung, and Pingtung)	44%
	Department of Communication and Design	0%		Eastern Taiwan (Yilan, Hualien, and Taitung)	4%
	Department of Tourism and Hospitality	19%		Offshore islands	18%
	Other departments	37%		Foreign student	1%

### Measurement model analysis

Before the statistical analysis of the structural equation model was conducted, the reliability and validity of the questionnaire results were tested to make the subsequent statistical analysis smoother. The analysis items included reliability analysis and validity analysis.

#### Reliability analysis

(1) General reliability indicators: To verify the reliability of each item, a reliability analysis (Cronbach’s α) was conducted for each dimension. In the second-order dimensions, the α values of leisure involvement and leisure benefits were 0.959 and 0.950, respectively; the α values of the first-order dimensions ranged from 0.833 to 0.959, which were above the minimum values of each threshold, and were within the high-reliability range suggested by Nunnally, as shown in Table 2.

**TABLE 2:** Measurement model parameter estimation.

The second-order dimensions	The first-order dimensions	Item	Factor loading (Out loading)	Cronbach’s α	CR value	AVE value
	Perceived usefulness	PU01	0.875	0.931	0.951	0.829
		PU03	0.920			
		PU05	0.935			
		PU06	0.910			
	Perceived ease of use	PEOU01	0.882	0.935	0.950	0.793
		PEOU02	0.892			
		PEOU03	0.892			
		PEOU04	0.900			
		PEOU05	0.887			
Leisure involvement				0.959	0.959	0.963
	Attractiveness	ATT01	0.899	0.938	0.951	0.765
		ATT02	0.893			
		ATT03	0.854			
		ATT04	0.829			
		ATT05	0.892			
		ATT06	0.847			
	Centrality	CN01	0.886	0.833	0.900	0.750
		CN02	0.865			
		CN03	0.878			
	Self-performance	SP01	0.897	0.936	0.949	0.758
		SP02	0.838			
		SP03	0.845			
		SP04	0.907			
		SP05	0.857			
		SP06	0.901			
	Intention to use	IN01	0.907	0.910	0.937	0.788
		IN02	0.836			
		IN05	0.904			
		IN06	0.898			
Leisure benefits				0.950	0.951	0.956
	Physiological benefits	PH01	0.918	0.912	0.938	0.792
		PH02	0.849			
		PH03	0.797			
		PH05	0.871			
	Psychological benefits	HE01	0.854	0.916	0.935	0.706
		HE02	0.846			
		HE03	0.840			
		HE04	0.832			
		HE05	0.869			
		HE06	0.886			
		HE07	0.893			
		HE08	0.887			
	Social benefits	SO01	0.875	0.907	0.935	0.781
		SO02	0.920			
		SO04	0.935			
		SO06	0.910			

(2) Composite reliability: To verify the consistency of the observed variables of each dimension, the composite reliability test was conducted. The results show that the CR values of leisure involvement and leisure benefits were 0.959 and 0951, respectively, for the second-order dimensions; the CR values of the first-order dimensions ranged from 0.900 to 0.959 and were above the threshold of 0.7 (Chin, 1998), as shown in Table 2.

(3) Reliability: Considering the factor loading value of 0.7 as the threshold value (Hair et al., 1998) for deleting items, we deleted the items PEOU02, PEOU06, CN04, CN06, IN04, PH06, PH07, and HE09 in order; the factor loading values of the remaining items were between 0.829 and 0.935. The reliability of this questionnaire is good, as shown in Table 2.

#### Construct validity analysis

(1) Convergent validity: The purpose of convergent validity is to ensure the consistency of each dimension. The average variance extracted (AVE) values of the second-order dimensions after the test were 0.963 and 0.956, respectively, for leisure involvement and leisure benefits; the AVE values of the first-order dimensions ranged from 0.706 to 0.963, which were all higher than the threshold value of 0.5 suggested by Fornell and Larcker (1981), as shown in Table 2. This finding indicated that the average explanatory power of each dimension exceeded 50% of the indicator, implying good convergent validity.

(2) Discriminant validity: One of the prerequisites in conducting the SEM analysis is to check the discriminant validity of the dimensions, in which the most commonly used items to check the discriminant validity are the cross-loadings and Formell–Larcker criterion, respectively. Table 3 shows that the factor loadings of the second-order and first-order dimensions were greater than the cross-loadings between the dimension and the other dimensions, shown as the bold values. According to the Formell–Larcker criterion, there is discriminant validity if the AVE square root of each dimension is greater than the correlation coefficient between the dimension and other dimensions. The research results show that the AVE square root of each dimension was between 0.814 and 0.911, which was greater than the correlation coefficient between each dimension and the other dimensions, as shown in Table 4, indicating good discriminant validity for all dimensions. Therefore, all the dimensions in this study had good construct validity.

**TABLE 3:** Cross-loadings of all dimensions in scale.

Dimension item	INV	BEN	PU	PEOU	ATT	CN	SP	IN	PH	HE	SO
PU01	0.415	0.444	**0.875**	0.618	0.373	0.422	0.363	0.586	0.365	0.408	0.421
PU03	0.406	0.436	**0.920**	0.674	0.335	0.401	0.393	0.514	0.388	0.402	0.385
PU05	0.411	0.406	**0.935**	0.651	0.357	0.411	0.377	0.544	0.332	0.385	0.365
PU06	0.418	0.427	**0.910**	0.636	0.366	0.395	0.392	0.542	0.351	0.396	0.399
PEOU01	0.519	0.481	0.586	**0.882**	0.490	0.479	0.457	0.567	0.370	0.452	0.464
PEOU02	0.505	0.520	0.656	**0.892**	0.459	0.465	0.464	0.583	0.406	0.496	0.483
PEOU03	0.504	0.506	0.604	**0.892**	0.440	0.492	0.466	0.552	0.386	0.478	0.485
PEOU04	0.459	0.469	0.657	**0.900**	0.401	0.451	0.423	0.532	0.375	0.449	0.424
PEOU05	0.473	0.519	0.649	**0.887**	0.428	0.462	0.422	0.602	0.423	0.476	0.499
ATT01	**0.816**	0.634	0.394	0.502	**0.879**	0.668	0.651	0.542	0.511	0.598	0.584
ATT02	**0.835**	0.604	0.305	0.415	**0.899**	0.641	0.691	0.489	0.469	0.580	0.558
ATT03	**0.830**	0.578	0.375	0.459	**0.893**	0.643	0.684	0.521	0.468	0.552	0.517
ATT04	**0.815**	0.586	0.349	0.418	**0.854**	0.612	0.705	0.513	0.479	0.556	0.528
ATT05	**0.751**	0.630	0.307	0.391	**0.829**	0.605	0.584	0.518	0.518	0.582	0.593
ATT06	**0.820**	0.618	0.332	0.428	**0.892**	0.658	0.654	0.506	0.500	0.569	0.592
CN01	**0.719**	0.670	0.411	0.454	0.582	**0.847**	0.637	0.534	0.548	0.613	0.643
CN02	**0.749**	0.683	0.401	0.457	0.657	**0.886**	0.613	0.549	0.551	0.653	0.614
CN03	**0.768**	0.655	0.353	0.459	0.654	**0.865**	0.672	0.506	0.477	0.663	0.575
SP01	**0.840**	0.642	0.378	0.461	0.704	0.679	**0.878**	0.546	0.484	0.631	0.580
SP02	**0.837**	0.649	0.364	0.442	0.680	0.675	**0.897**	0.552	0.481	0.639	0.592
SP03	**0.779**	0.560	0.402	0.442	0.642	0.608	**0.838**	0.468	0.433	0.535	0.520
SP04	**0.772**	0.596	0.364	0.389	0.618	0.606	**0.845**	0.479	0.443	0.571	0.570
SP05	**0.825**	0.619	0.347	0.447	0.657	0.650	**0.907**	0.521	0.441	0.609	0.581
SP06	**0.800**	0.594	0.336	0.434	0.653	0.644	**0.857**	0.468	0.413	0.576	0.583
IN01	0.607	0.636	0.517	0.594	0.564	0.589	0.529	**0.901**	0.548	0.598	0.555
IN02	0.607	0.639	0.521	0.558	0.557	0.592	0.534	**0.907**	0.511	0.617	0.569
IN05	0.500	0.515	0.536	0.548	0.443	0.458	0.472	**0.836**	0.428	0.489	0.455
IN06	0.571	0.576	0.563	0.562	0.517	0.522	0.527	**0.905**	0.453	0.559	0.515
PH01	0.503	**0.720**	0.322	0.368	0.470	0.507	0.426	0.444	**0.897**	0.556	0.577
PH02	0.519	**0.726**	0.354	0.404	0.490	0.535	0.429	0.492	**0.894**	0.559	0.595
PH03	0.593	**0.800**	0.382	0.429	0.551	0.598	0.505	0.550	**0.918**	0.666	0.631
PH05	0.530	**0.741**	0.345	0.364	0.480	0.514	0.475	0.460	**0.849**	0.620	0.581
HE01	0.527	**0.761**	0.370	0.408	0.474	0.536	0.461	0.502	0.640	**0.754**	0.617
HE02	0.628	**0.800**	0.414	0.475	0.571	0.642	0.544	0.588	0.583	**0.844**	0.641
HE03	0.662	**0.808**	0.411	0.495	0.582	0.655	0.604	0.585	0.571	**0.853**	0.663
HE04	0.591	**0.701**	0.336	0.408	0.510	0.549	0.568	0.491	0.489	**0.763**	0.544
HE05	0.586	**0.785**	0.338	0.427	0.539	0.606	0.498	0.491	0.548	**0.832**	0.650
HE06	0.678	**0.789**	0.340	0.436	0.565	0.679	0.646	0.514	0.540	**0.852**	0.632
HE07	0.618	**0.780**	0.307	0.408	0.549	0.608	0.562	0.523	0.522	**0.831**	0.656
HE08	0.568	**0.711**	0.325	0.375	0.462	0.550	0.564	0.465	0.505	**0.774**	0.543
SO01	0.626	**0.767**	0.426	0.483	0.580	0.587	0.557	0.530	0.601	0.639	**0.868**
SO02	0.622	**0.783**	0.379	0.451	0.563	0.616	0.550	0.540	0.584	0.670	**0.885**
SO04	0.640	**0.790**	0.320	0.431	0.556	0.637	0.588	0.476	0.574	0.685	**0.894**
SO06	0.663	**0.805**	0.400	0.505	0.570	0.648	0.621	0.547	0.611	0.697	**0.887**

The factor loadings of the second-order and first-order dimensions were greater than the cross-loadings between the dimension and other dimensions, show as the bold values.

**TABLE 4:** Discriminant validity test of all dimensions of scale.

Dimension	Formell–Larcker								
	
	PU	PEOU	ATT	CN	SPO	IN	PH	HE	SO
PU	0.911								
PEOU	0.708	0.891							
ATT	0.393	0.498	0.875						
CN	0.448	0.527	0.730	0.866					
SPO	0.419	0.501	0.758	0.740	0.871				
IN	0.601	0.637	0.588	0.611	0.582	0.888			
PH	0.395	0.441	0.560	0.606	0.516	0.548	0.890		
HE	0.437	0.528	0.654	0.743	0.683	0.640	0.676	0.814	
SO	0.431	0.529	0.642	0.704	0.656	0.592	0.762	0.762	0.884

In summary, the measurement model of this study was tested for internal consistency (composite reliability), general indicator reliability, convergent validity (average variance), and discriminant validity; all results met the academic requirements. This finding indicated that the measurement systems of the dimensions by “ease of use of water facilities,” “usefulness of water facilities,” “water leisure involvement,” “intention to participate in water activities,” and “water leisure benefits” of this scale had good reliability, convergent validity, and discriminant validity. Thus, the structural model analysis can be conducted to examine the causal pathways among these dimensions.

### Structural model

According to the systematic approach to structural model evaluation proposed by Hair et al. (2016), the process is divided into stages, namely, collinearity diagnosis of the structural model, significance test of path coefficients, evaluation of *R*^2^ size, and assessment of the explanatory effect value *f*^2^.

#### Collinearity assessment analysis

From the Inner variance inflation factor (VIF) Values, we can observe that the VIF values among the relevant dimensions were all less than the threshold value of 5, indicating that the collinearity problem among the dimensions in the structural model was not serious. Therefore, the collinearity problem would not adversely affect the estimation of the path coefficients of the structural model. The test results are shown in Tables 5, 6. After the items—PU04, CN05, IN03, PH04, SO03, and SO05—were deleted, the values of the Inner VIF among the dimensions were between 1.000 and 2.321, which were within the range of values suggested by Hair et al. (2016). Therefore, the structural model of this study did not have a collinearity problem, as shown in Table 5.

**TABLE 5:** Inner variance inflation factor (VIF) test of structural model.

	PU	PEOU	INV	IN	BEN
**PU**					
**PEOU**	1.000				
**INV**	2.007	2.007			
**IN**	2.030	2.321	1.455		
**BEN**			1.714	1.714	

**TABLE 6:** Structural model evaluation.

Hypothesis	Relationship	Path coefficient	*t-*value	Decision*	*R* ^2^	*f* ^2^	95%CI LL	95%CI UL	Fit
H1	PEOU → PU	0.708	22.896	Supported	0.502	1.007	0.656	0.758	SRMR = 0.069 NFI = n/a RMS_Theta = 0.145
H2	PEOU → IN	0.239	4.076	Supported	0.561	0.056	0.141	0.339	
H5a	PU → IN	0.250	4.140	Supported		0.070	0.149	0.347	
H6a	INV → IN	0.400	10.304	Supported		0.251	0.335	0.466	
H3	PEOU → INV	0.464	6.994	Supported	0.313	0.156	0.357	0.573	
H4a	PU → INV	0.124	1.785	Not Supported		0.011	0.013	0.235	
H7a	IN → BEN	0.287	5.614	Supported	0.643	0.134	0.200	0.369	
H8	INV → BEN	0.586	11.668	Supported		0.561	0.503	0.669	

“*” means it is significant at the significance level of 0.05.

#### Test of path relationship

**FIGURE 2:**
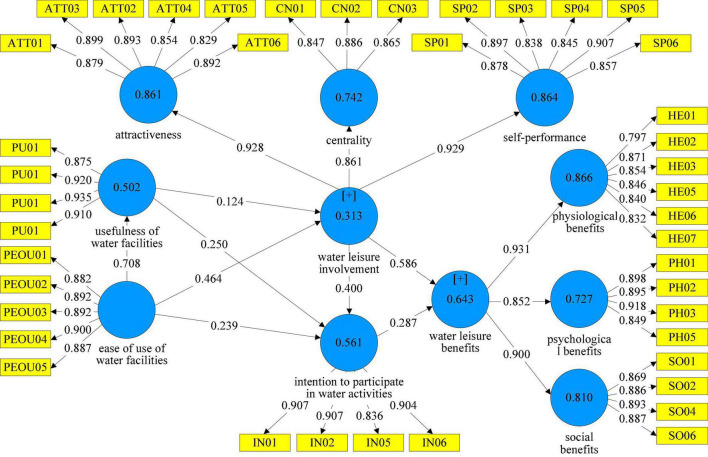
Structural model analysis results.

In this study, SmartPLS3 was used to conduct the path analysis among the dimensions of the study structure. The path analysis was performed by bootstrapping with 5,000 times of repeated data sampling, and the research hypotheses were tested. Given that the study structure was a single clear directional relationship, a two-tailed test was adopted by this study with a significance level (*p*-value) less than 0.05 (Bassellier and Benbasat, 2004) as the judgment criterion, as shown in Figure 2. The path coefficients of the hypothesized relationships—H1, H2, H3, H5a, H6a, H7a, and H8—were 0.464, 0.708, 0.287, 0.586, 0.400, 0.239, and 0.250, respectively, all of them reaching the significant level. All hypotheses were supported except for H4a, as shown in Table 6.

#### Evaluation of model explanatory power and explanatory effect value

The explanatory power of the ease of use of water facilities and the usefulness of water facilities on water leisure involvement in this model was 0.313, which is a weak explanatory power according to the criteria. For other dimensions, the explanatory power of the ease of use of water facilities for the usefulness of water facilities was 0.502; the explanatory power of water leisure involvement and the intention to participate in water activities for water leisure benefits was 0.643; the explanatory power of the ease of use of water facilities, the usefulness of water facilities and water leisure involvement for the intention to participate in water activities was 0.561, which were all moderate explanatory power, as shown in Table 6.

The explanatory power of the external dimension “ease of use of water facilities” on the internal dimension “intention to participate in water activities” was 0.056; the explanatory power of the external dimension “usefulness of water facilities” on the internal dimension “intention to participate in water activities” was 0.070; the explanatory power of the outer derivative “intention to participate in water activities” on the inner derivative “water leisure benefits” was 0.134. They were all small explanatory power. The explanatory power of the outer derivative dimension “water leisure involvement” on the inner derivative dimension “intention to participate in water activities” was 0.251, and the explanatory power of the outer derivative dimension “ease of use of water facilities” on the inner derivative dimension “water leisure involvement” was 0.156. Both were medium effect explanatory power. The explanatory effect value of the external dimension “water leisure involvement” for the internal dimension “water leisure benefits” was 0.561, and the explanatory of the external dimension “ease of use of water facilities” for the internal dimension “usefulness of water facilities” was 1.007. Both were a large effect explanatory power. Overall, the explanatory power of the external dimensions to the internal dimensions was above medium to high, as shown in Table 6.

#### Overall model fit evaluation

The model test result of SRMR = 0.069 < 0.08 indicated that the model fit was good; although RMS_theta = 0.145 > 0.12, the difference was insignificant. Therefore, the model’s overall fit was good, meeting its academic requirement, as shown in Table 6.

In summary, as far as the quality of the structural model is concerned, the model’s explanatory power, predictive ability, or overall goodness of fit index were all in line with the academic requirements for model quality. Therefore, the conceptual model constructed in this study and the causality of each latent variable to both theoretical and practical applications is valuable.

### Test of mediating effect

Following the analysis of the overall structural model, this study investigated the mediating roles of “usefulness of water facilities,” “water leisure involvement,” and “intention to participate in water activities” in the overall model, as shown in Table 7, The results of this test are as follows:

**TABLE 7:** Test of mediating effect.

Independent variable	Mediating variable	Dependent variable	Direct effect	Indirect effect	Overall effect	VAF	Hypotheses
INV	IN	BEN	0.586* (11.466)	0.115* (5.409)	0.701	16.37%	H7b was supported
PEOU	PU	INV	0.464* (7.040)	0.050 (1.753)	0.552	15.93%	H4b was not supported
PEOU	INV	IN	0.239* (4.056)	0.186* (5.776)	0.425	43.74%	H6b was supported
PU	INV	IN	0.250* (4.148)	0.050 (1.746)	0.300	16.57%	H6b′ was not supported
PEOU	PU	IN	0.239* (4.056)	0.177* (4.011)	0.416	42.59%	H5b was supported

“*” means it is significant at the significance level of 0.05; () means the *t*-value.

#### Mediating role of dimension of usefulness of water facilities

(1) The usefulness of water facilities showed a significant mediating effect on the ease of use of water facilities and the intention to participate in water activities.

In H7b, the “usefulness of water facilities” showed a significant mediating effect on the “ease of use of water facilities” and the “intention to participate in water activities,” with an indirect effect value of 0.177 and *t*-value of 4.022; hence, the hypothesis was supported. The VAF value of 42.59% represented a partial mediating effect of the usefulness of water facilities.

(2) The usefulness of water facilities did not have a mediating effect on the ease of use of water facilities and water leisure involvement.

In H4b, the “usefulness of water facilities” did not have a significant mediating effect on the “ease of use of water facilities” and “water leisure involvement,” with an indirect effect value of 0.050, a *t*-value of 0.050, and a *t*-value of 1.753; hence, the hypothesis was not supported.

#### Mediating role played by dimension of water leisure involvement

(1) Water leisure involvement played a mediating role in the ease of use of water facilities and the intention to participate in water activities.

In H6b, there was a significant mediating effect of “water leisure involvement” on the “ease of use of water facilities” and the “intention to participate in water activities,” with an indirect effect value of 0.186 and a *t*-value of 5.786; hence, the hypothesis was supported. The VAF value was 43.74%, which meant that the usefulness of water facilities had a partial mediating effect.

(2) The relationship between the usefulness of water facilities and the intention to participate in water activities was mediated by water leisure involvement.

In H6b′, “water leisure involvement” did not have a significant mediating effect on the “usefulness of water facilities” and the “intention to participate in water activities,” and its indirect effect value was 0.050 and the *t*-value 1.746; hence, the hypothesis was not supported.

#### Mediating role of dimension of intention to participate in water activities

In H7b, the “intention to participate in water activities” had a significant mediating effect on “water leisure involvement” and “water leisure benefits,” with an indirect effect value of 0.115 and a *t*-value of 5.409; hence, the hypothesis was supported. The VAF value of 16.37% represented only a weak partial mediating effect of the intention to participate in water activities.

### Interference

**FIGURE 3:**
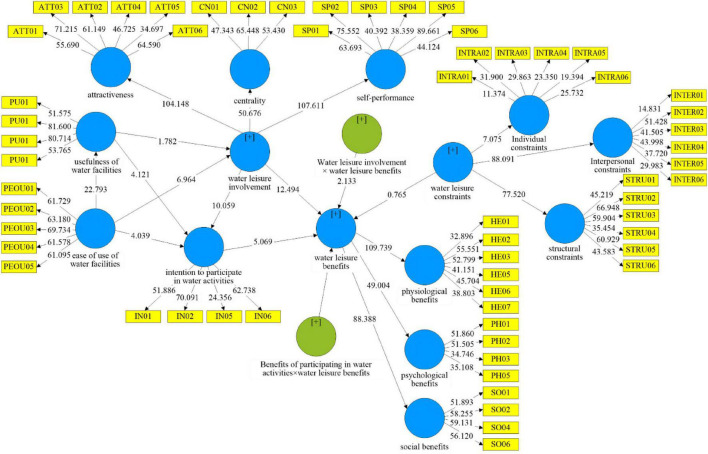
Interference model analysis result.

This study investigated whether water leisure benefits are interfered with by factors besides the mediating effect of the intention to participate in water activities. After reviewing the relevant literature, we examined whether water leisure constraints could be an interfering factor between water leisure involvement and the intention to participate in water activities on water leisure benefits, as shown in Figure 3.

After the interference effect analysis, water leisure constraints had an interference effect between water leisure involvement and water leisure benefits, but not between the intention to participate in water activities and water leisure benefits, as shown in Table 8:

**TABLE 8:** Test of interference effect.

Hypotheses	Path	Coefficient value	*t-*value	Test result
H9	INV × Barriers → BEN	–0.118	2.102	was supported
H10	IN × Barriers → BEN	0.052	0.842	was not supported

**FIGURE 4:**
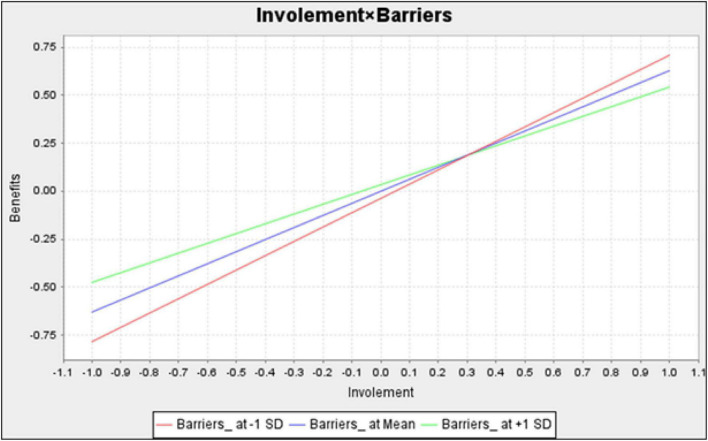
Simple slope analysis of interaction term “water leisure involvement × water leisure constraints.”

1. In the interaction term “water leisure constraints × intention to participate in water activities,” the interference effect on water leisure benefits was 0.052, and the *t*-value was 0.842, which was less than 1.65 and insignificant. Therefore, this study concluded that water leisure constraints failed to interfere with the intention to participate in water activities and water leisure benefits.
2. In the interaction term “water leisure involvement x water leisure constraints “, the effect of interference on water leisure benefit was –0.118 with a *t*-value of 2.102, which was greater than 1.65 and significant. Therefore, it is inferred that water leisure constraints interfered with the intention to participate in water activities and water leisure benefits. The simple slope analysis of Figure 4 clearly shows that the lines on the simple slope analysis plot were not parallel or even intersecting, indicating that the interference effect of water leisure constraints on the path of “water leisure involvement → water leisure benefits” was significant.

The simple slope analysis in Figure 4 shows that the slope of the water leisure constraints was greater when the water leisure constraints were low (green line) than when the water leisure involvement was high (blue line) for a mean of positive and negative one standard deviation, respectively. This finding indicated that the path relationship of “water leisure involvement → water leisure benefits” was weaker when the water leisure constraints were low. Meanwhile, the path relationship of “water leisure involvement → water leisure benefits” was stronger when the water leisure constraints were high.

## Conclusion and suggestions

### Research conclusion

The results of the structural model path analysis imply that most hypotheses in this study were supported, which are in line with expectations. The descriptions are as follows:

#### Relationship between the technology acceptance model and various dimensions

(1) Relationships among the ease of use of water facilities, water leisure involvement, and the intention to participate in water activities.

The results of the study showed that the “ease of use of water facilities” had a positive effect on the predictions of “water leisure involvement” and the “intention to participate in water activities” in the use of online digital teaching materials for assisted teaching. In addition to verifying that college students have had a substantial foundation in the ability to use information devices for learning, it also verified that college students felt that using digital teaching materials as a learning aid tool could not only increase their intention to participate in water activities, but also increase their investment in water leisure activities. Hence, H2 and H3 were both verified.

(2) Relationships among usefulness of water facilities, water leisure involvement, and intention to participate in water activities.

This study found that students valued the ease of access to online digital materials and the accuracy and usefulness of the browsing and the learning information provided. Therefore, teachers should continue refining online materials and learning information for teaching online to increase students’ willingness to use digital materials for independent learning. The results show that H4a was not supported, while H5a was supported. This finding indicated that, although digital teaching aids can attract students’ willingness to participate in the course, if the instructor does not continue to provide incentives or aids to attract learners to online teaching, it will not enhance students’ engagement and enjoyment of learning at the moment.

(3) The ease of use of water facilities had a positive effect on the usefulness of water facilities.

The experimental results show that H1 was supported, which also confirmed that the “ease of use of water facilities” had a highly positive effect on the “usefulness of water facilities,” indicating that the effect of ease of use on the usefulness of the TAM can be positively verified for all types of technology integration experience activities.

#### Relationship among dimensions of water leisure involvement, intention to participate in water activities, and water leisure benefits

(1) Water leisure involvement had a positive effect on the intention to participate in water activities and water leisure benefits.

The result of H6a showed that the more the learners were involved in the course, the more they paid attention to and participated in the information and activities related to water leisure; hence, the more they were willing to participate in the course. This result is consistent with the study of Meng and Choi (2018). The research result that H8 was supported showed that the deeper the learners’ involvement in the digital learning course, the more their feeling and experience of course activities could improve the physical and mental state of the course participants and meet their personal needs, showing a high level of leisure benefits and more positive behavioral performance of participation.

(2) The intention to participate in water activities had a positive effect on water leisure benefits.

The literature review has shown that leisure benefits influence the intention to participate. The research results indicated that the H7a was supported, which showed that the stronger the intention of the learners to participate in the course, the more they would experience the information and activities related to water leisure, allowing students to gain physical or psychological satisfaction from the course activities to enhance their social skills.

#### Mediating effect of the technology acceptance model on water leisure involvement and intention to participate in water activities

(1) The usefulness of water facilities mediated the ease of use of water facilities and the intention to participate in water activities.

In the research results, H5b was supported. This finding showed that the easier it was to understand and use the content and operation of online teaching aids, the more enjoyable the learning process was for students, and the easier they found to use them, which in turn would increase their intention to participate in water activities—that is, the features that help users to use can increase the intention to participate in water activities.

(2) The usefulness of water facilities did not play a mediating role between the ease of use of water facilities and water leisure involvement.

The research results show that H4b was not supported. This finding indicated that even if the content or operation of online teaching aids were easy to understand and easy to use, they could not attract students’ intention to engage in water leisure activities. The reason behind this phenomenon is that water leisure is a dynamic activity, and the online course of dynamic simulation due to COVID-19 cannot attract students to participate in the experience.

#### Mediating effect of water leisure involvement in the relationship between usefulness/ease of use of water facilities and intention to participate in water activities

(1) Water leisure involvement played a mediating role in the relationship between the ease of use of water facilities and the intention to participate in water activities.

The research results show that H6b was supported, implying that the easier it was to understand and use the content or operation of online teaching aids, the more students would engage in the experience of water activities through the aid of tools that help them to use them easily to increase their intention to participate in water activities.

(2) Water leisure involvement did not have a mediating role between the usefulness of water facilities and the intention to participate in waterside activities.

H6b′ was not supported in the research result, indicating that the ease of use of the content or operation of online teaching aids could not attract students’ behavioral intention to participate in water leisure activity experiences. This phenomenon results from the fact that if the online courses are organized or taught in the same way, they will not be able to attract students to engage in leisure activities, and students will no longer be willing to participate in the activities.

#### Intention to participate in water activities mediated the relationship between water leisure involvement and water leisure benefits

The research results indicated that H7b was supported. This finding showed that if the online courses could attract students to participate in learning, it would be reflected in the students’ increased interest and participation in the information and activities of various water activities. In addition, it could increase their physiological and psychological satisfaction benefits.

#### Interference effect of water leisure constraints on leisure benefits hindered by water leisure involvement/intention to participate in water activities

(1) The interaction term “water leisure involvement × water leisure constraints” had an interference effect on water leisure benefits.

The research results supported H9, indicating that the water leisure constraints interfered with water leisure involvement and water leisure benefits. When students have a high tendency to participate in online courses, they may not be able to fully immerse themselves in the activity experience due to the lack of information equipment and hardware or the limitation of the activity area, depriving them from spiritual help.

The simple slope analysis results show that the straight lines of the simple slope analysis plot were not parallel or even intersected, indicating that the path coefficient of “water leisure involvement → water leisure benefits” was negative (–0.118), and the interference effect was significant. We found that the path of “water leisure involvement → water leisure benefits” was stronger in the initial stage of the course when the students’ perceived benefits were low. As the course progressed, the students’ perceived benefits increased, and the path of “water leisure involvement → water leisure benefits” became weaker. This finding means that when the students’ perceived benefits are low, the teacher should focus on teacher-student or student interaction to stimulate and attract students’ continuous participation in the course to enhance their benefits positively.

(2) The interaction term “intention to participate in water activities × water leisure constraints” did not interfere with water leisure benefits.

The research result supported H10, indicating that water leisure constraints did not interfere with the intention to participate in water leisure activities and water leisure benefits. When students overcome strong barriers, their expectations of participating in leisure experience activities become higher. However, when the expectations are too high, it can easily lead to a gap in the expectations and lower the overall benefits for the students to reevaluate whether the benefits of overcoming the obstacles are worthwhile. The benefits will not exist when the students believe the benefits are disproportionate to the efforts made to overcome the obstacles.

Research findings mentioned above prove that online digital approaches used to support the teaching of water leisure activities actually make student learning more effective in the post-COVID-19 era. Students’ mindset and intentions are further explored through ease of use and usefulness proposed by the Technology Acceptance Model. Through these findings, teachers can keep learners more willing to be involved in courses and make them more concentrated on courses by deploying the interactional design of textbooks and availability of learning platforms, thus achieving expected learning effectiveness. Constant innovations should be made for textbooks relating to water recreation and teaching supporting facilities to keep students involved in the long term. As the pandemic presses a pause button on water leisure activities, college students accustomed to mobile learning can make full use of digital textbooks to facilitate their acquisition of water activities. In addition, teachers should also make flexible use of digital textbooks to design and improve their teaching contents, thus strengthen students’ enthusiasm about being involved and engaged in water activities and even extend such willingness. A stronger willingness of engagement would have a positive effect on the effectiveness of water leisure activities, by satisfying their physical and psychological demands and improving their social capabilities. Therefore, mobile learning, when applied to water leisure activities, can appease lovers of water leisure activities for the failure to engage in these activities in the post-COVID-19 era.

### Research recommendations and implications

Base on the above conclusion, the results of this study indicated that digital learning-assisted courses could not help in consistently engaging students in the courses. Therefore, the following course recommendations are made to shed light on the implementation of online teaching in the post-pandemic era for subsequent studies:

#### Curriculum

(1) Proximity course: Relevant video and somatosensory games shall be integrated into the course to improve student involvement.

(2) Remote course: An interactive water activity somatosensory course across domains shall be developed to correct students’ posture to enhance their intention to participate in the course.

#### Teaching

(1) Interactivity

This study verified that usefulness affected users’ intention to learn. Thus, it is a direction that we should work on to improve the interactivity of users’ cognitive digital learning.

(2) Fun

Teachers may organize or develop interesting teaching materials for students to download or interact online or plan interesting questions for discussion or prize-winning questions for students to exchange and discuss.

#### Research

This study proposed a theoretical model applicable to the water leisure context to explain students’ participation intentions, which are seldom explored. Secondly, the TAM’s focus on new water leisure techniques combined the TAM’s water leisure participation and benefits to gain valuable insights into students’ intentions to participate in water leisure activities. In the water leisure setting, this study demonstrated that water leisure constraints neither mediated the relationship between involvement and intention nor between benefits and intention. Moreover, the study found theoretical relevance of perceived usefulness, involvement, and benefits in mediating the relationship between perceived ease of use and students’ intention to participate in water leisure activities.

## Data availability statement

The raw data supporting the conclusions of this article will be made available by the authors, without undue reservation.

## Ethics statement

Ethical review and approval was not required for the study on human participants in accordance with the local legislation and institutional requirements. Written informed consent for participation was not required for this study in accordance with the national legislation and the institutional requirements.

## Author contributions

K-CC: conceptualization, formal analysis, and writing—original draft. K-CC and S-JL: data curation and investigation. C-CT: supervision. K-CC, S-JL, and C-CT: writing—review and editing. All authors have read and agreed to the published version of the manuscript.
